# Association between mild cognitive impairment and sleep quality in patients with chronic heart failure: a cross-sectional study

**DOI:** 10.3389/fpsyt.2025.1704672

**Published:** 2025-11-10

**Authors:** Yanmei Gan, Tingting Liao, Yao Du, Lingfang Liu, Lan Luo, Wenhua Huang, Gaoye Li

**Affiliations:** 1 Department of Cardiovascular Medicine, The First Affiliated Hospital of Guangxi Medical University, Nanning, China; 2 Department of Radiation Oncology, The First Affiliated Hospital of Guangxi Medical University, Nanning, China

**Keywords:** chronic heart failure, mild cognitive impairment, sleep quality, sleep disorders, risk factors

## Abstract

**Objective:**

Mild cognitive impairment (MCI) has increasingly been recognized as a significant comorbidity in patients with chronic heart failure (CHF), adversely affecting prognosis and quality of life, despite limited research examining the role of sleep quality in this relationship. This study aimed to assess the prevalence of MCI and poor sleep quality in patients with CHF and to examine the association between them.

**Methods:**

We conducted a cross-sectional study among 329 patients with CHF recruited from a hospital in Nanning, China, between September 2024 and June 2025. We collected the sociodemographic and clinical characteristics from all participants using a general information questionnaire. We assessed global cognitive function with the Beijing version of the Montreal Cognitive Assessment Scale (MoCA-BJ) and evaluated subjective sleep quality over the preceding one-month period using the Pittsburgh Sleep Scale Index (PSQI). We examined the association between MCI and sleep quality using point-biserial correlation coefficient analysis, and then further assessed it with hierarchical regression models, adjusting for potential confounders.

**Results:**

The study revealed high prevalence rates of both MCI (58%) and poor sleep quality (70.52%) in the CHF population. A significant positive correlation was identified between MCI and poor sleep quality (*r* = 0.322, *P* < 0.01). Multivariable analysis demonstrated that sleep quality remained independently associated with MCI after adjusting for other risk factors, with the final model explaining over half of the variance in MCI risk.

**Conclusion:**

Poor sleep quality shows a strong independent association with MCI in CHF patients. These findings highlight the importance of sleep assessment in CHF management and suggest that addressing poor sleep quality may represent a valuable approach in comprehensive care strategies aimed at preserving cognitive function in this CHF population.

## Introduction

Chronic heart failure (CHF) is a complex syndrome of inadequate cardiac output due to cardiac dysfunction, typically presenting with symptoms such as shortness of breath and peripheral edema (1). The global burden of CHF is escalating, driven by an aging population and the growing prevalence of risk factors such as hypertension, obesity, and diabetes (2). The American Heart Association reported in its 2022 statistics that over 64.3 million individuals worldwide were living with CHF (3), representing approximately 1% to 2% of the global population (4). Furthermore, the prevalence of CHF is projected to increase by 46% by 2030 (5), highlighting a notable projected growth and its substantial expanding public health challenge. The clinical burden is accompanied by a substantial economic one, with CHF estimated to cost the global economy up to $108 billion annually in both direct medical expenses and indirect costs (6). Consequently, this trend continues to place sustained pressure on healthcare systems worldwide.

Given the significant clinical and economic burden of CHF, it is imperative to understand its associated comorbidities, particularly cognitive impairment. Cognition refers to the neuropsychological processes involved in constructing mental representations from the perception, processing, and synthesis of external information, which subsequently enables the acquisition of applicable knowledge (7). Cognitive impairment is an acquired and persistent clinical syndrome marked by deterioration in cognitive domains (8). This decline results in reduced capacity for daily activities and occupational functioning, and can also lead to behavioral alterations (9). Notably, mild cognitive impairment (MCI)represents a transitional state between normal cognitive aging and dementia, with a conversion rate to dementia of 10% to 15% per year (10).

Patients with CHF exhibit an elevated prevalence of MCI compared with the general population, with rates reported to be 1.5 to 2 times higher (11). A multicenter study in Italy showed that the prevalence of MCI among 1,511 hospitalized patients with CHF was 35% (12). Similarly, a Singaporean cohort study of 100 patients with chronic compensated CHF found a prevalence of 78% for MCI (13). Collectively, these findings demonstrate a high prevalence of MCI among individuals with CHF, which substantially affects their quality of life. Furthermore, established risk factors and clinical correlates for MCI in this patient population include age, lower educational level, BMI, nutritional status, and systemic inflammatory markers (14–16). Therefore, early detection of cognitive decline in CHF patients followed by timely intervention may help stabilize cognitive function slow its deterioration and improve overall quality of life.

Beyond cognitive impairment, another highly prevalent and interconnected comorbidity in patients with CHF is sleep disorders. Sleep disorders are defined as disruptions in the sleep-wake cycle, encompassing abnormalities in sleep quality, duration, or timing, along with undesirable behavioral or physiological events (17). Common manifestations include insomnia, circadian rhythm sleep-wake disorders, night terrors, and nightmares (18). It is estimated that approximately 75% of patients with CHF experience sleep disorders (19). By exacerbating autonomic dysfunction, accelerating disease progression, and impairing cardiac function, these disorders thereby significantly diminish patients’ quality of life (20). In addition, sleep disorders contribute to cognitive deficits through disruptions in neural plasticity synaptic communication and neurotransmitter balance (21). A quantitative meta-analysis further indicated that sleep initiation difficulties insufficient sleep duration and daytime sleepiness contribute to cerebral beta-amyloid deposition thereby elevating cognitive impairment risk (22, 23). While its cross-sectional design precludes causal inference, a large sample community-based study further supports the association between sleep disorders and MCI risk (24).

Although previous studies have linked poor sleep quality to MCI risk, limited research has directly investigated this relationship within the specific CHF population. Therefore, this study aims to clarify the correlation between MCI and sleep quality in patients with CHF to inform the development of targeted interventions for improving prognosis.

## Materials and methods

### Study participants and design

We conducted a cross-sectional study and recruited 329 patients with CHF from the Department of Cardiovascular Medicine of a tertiary hospital in Nanning, Guangxi, China, between September 2024 and June 2025. We identified potential participants by screening the hospital HIS for chronic heart failure diagnoses. Eligible patients who met all criteria were approached for participation.

The inclusion criteria were as follows: (1) Diagnosis of CHF according to the "Chinese Guidelines for the Diagnosis and Treatment of Heart Failure 2024"(25); (2) Age≥18 years; (3) Willingness to participate in the study. 

The exclusion criteria were as follows: (1) Diagnosis of dementia or other major neurocognitive disorders; (2) Use of psychotropic or antiepileptic medications; (3) Presence of severe neurological conditions (such as Parkinson's disease, stroke with significant functional impairment, or other neurodegenerative disorders); (4) Any condition that would preclude cooperation with study protocols.

### Sample size

The sample size was determined using G*Power 3.1 version (26). Based on a logistic regression model, the calculation assumed an α error probability of 0.05, a statistical power of 0.95, 24 predictor variables, and an expected effect size *R²* of 0.15. The minimum sample size required was calculated to be 277 cases. Considering the 10% data instability, it was finally determined to include 329 patients.

### Measurement methods

#### General information questionnaire

It includes the following contents (1):General demographic information of the patient: age, sex, BMI, educational level, marital status (2);Disease-related data: smoking history, drinking history, NYHA class, hypertension, diabetes, stroke, atrial fibrillation (3);Examination report and laboratory indicators: Hemoglobin, high-sensitivity C-reactive protein(hs-CRP), total cholesterol, triglycerides (TG), serum creatinine (Scr), aspartate aminotransferase (AST), alanine aminotransferase (ALT), NT-ProBNP, left ventricular ejection fraction (LVEF).

#### The Beijing version of the Montreal cognitive assessment scale

The MoCA-BJ was originally developed by Nasreddine et al. (27) and subsequently translated and culturally adapted into Chinese by Yang et al. (28). It has been widely used in China for assessing cognitive impairment. The scale has 12 items with a total score of 30. The assessment covers eight cognitive domains, including memory, delayed recall, visuospatial/executive functions, naming, attention, language, abstraction, and orientation. Higher scores indicate better cognitive function. A score of ≥26 suggests normal cognition, 16–25 suggests MCI, and a score below 16 indicates severe cognitive impairment. For participants with ≤12 years of education, one point was added to the total score, with a maximum score of 30. The Cronbach’sα coefficient of this scale is 0.82.

#### Pittsburgh sleep quality index

The PSQI was developed by Buysse et al. (29) and translated by Chinese scholars Liu Xianchen et al. (30). It is used to assess the subjective sleep quality over the past month. The PSQI comprises 19 items categorized into seven subdomains: subjective sleep quality, sleep latency, sleep duration, habitual sleep efficiency, sleep disturbances, use of sleep medication, and daytime dysfunction. Each component is scored from 0 to 3, resulting in a global score that ranges from 0 to 21. A total score >7 is indicative of poor sleep quality. The scale has demonstrated good test-retest reliability and validity. The Cronbach’sα coefficient of this scale is 0.84.

#### Patient health questionnaire-9 depression scale

The scale was developed by American scholars Spitzer et al. (31) and translated into Chinese by Bian Cuidong et al. (32). It is an important tool for screening and assessing depressive symptoms, consisting of 9 items to evaluate the feelings of the subjects in the past two weeks. The total score ranges from 0 to 27 points. The degree of depressive symptoms is classified based on the total score into none (0–4 points), mild depressive symptoms(5–9 points), moderate depressive symptoms(10–14 points), moderate-to-severe depressive symptoms(15–19 points), and severe depressive symptoms(20–27 points). The Cronbach’sα coefficient of this scale is 0.771.

#### Generalized anxiety disorder scale

The GAD-7 scale was developed by Spitzer et al. (33). This scale has 7 items with a total score of 21. Anxiety symptoms is classified based on the total score as none (0–4 points), mild anxiety symptoms(5–9 points), moderate anxiety symptoms(10–14 points), and severe anxiety symptoms(15–21 points). The Cronbach’sα coefficient of this scale is 0.92 (34).

#### Controlling nutritional status score

The CONUT score was developed by de Ulibarri et al. (35) in 2005 as a tool for nutritional screening and monitoring. In recent years, it has been widely used among hospitalized patients with various chronic diseases such as cancer and cardiovascular diseases. This tool consists of three parameters: The total score is obtained by adding up the scores of serum Albumin concentration (ALB), peripheral lymphocyte count (LYM), and total cholesterol concentration (TC). The total score ranges from 0 to 12 points. The higher total score indicates a greater risk of malnutrition (36).

### Data collection

The researcher explained the purpose, significance, methods, and principles of confidentiality and anonymity pertaining to the study to each patient. Additionally, any questions raised by patients were addressed patiently by the researcher. Upon agreement from the patient, they scanned a QR code linked to a questionnaire for completion while receiving assistance from the researcher based on standardized instructions. The estimated time for filling out the questionnaire was approximately 15 to 20 minutes. After completion, researchers reviewed each questionnaire for errors or omissions and promptly clarified any uncertain responses with patients before submitting electronic forms to ensure accuracy. Furthermore, disease-related information along with biochemical test reports were collected from patients’ electronic medical records and cross-verified by two researchers to guarantee data reliability.

### Statistical analysis

The data were analyzed and organized using SPSS version 27.0 software. Continuous data are presented as mean ± standard deviation or median with interquartile range (M (P25, P75)), while categorical data are described in terms of frequency and percentage. The normality of all continuous variables was assessed using the Shapiro-Wilk test. As none of the variables followed a normal distribution (all *P* < 0.05), non-parametric tests were used throughout the analysis. Group comparisons were performed using the Mann-Whitney U test for continuous variables and the chi-square test for categorical variables. Variables with significant associations with MCI (*P* < 0.05) in univariate analyses were eligible for inclusion in the binary logistic regression. Only those that retained statistical significance in the multivariate model were retained in the final analysis.

To assess the independent role of sleep quality, two models were constructed utilizing hierarchical regression (block input method): Model 1 (the benchmark model) incorporated only covariates such as demographic characteristics and medical history; Model 2 built upon Model 1 by additionally including total sleep quality scores as the primary independent variable. The model fit for each hierarchical block was assessed and compared. The improvement in model fit from Model 1 to Model 2 was formally tested using the Likelihood Ratio Test (LRT). This test is based on the difference in the -2 Log-Likelihood (-2LL) values between the nested models, which follows a chi-square distribution with degrees of freedom equal to the number of new parameters added. A significant *P*-value for the LRT indicates that the new block of variables significantly improves the model’s fit to the data. By comparing changes in -2LL across both models and conducting likelihood ratio tests, we evaluated the independent contribution of sleep quality to predictive capabilities within these models. Regression results are reported as odds ratios (*OR*) along with their corresponding 95% confidence intervals (*CI*). *P* value < 0.05 was deemed statistically significant.

## Results

### Baseline characteristics

A total of 334 patients with CHF were investigated in this study, of whom 5 patients were invalid. Therefore, 329 valid data were ultimately collected for analysis. A total of 191 patients of CHF patients with MCI, a rate of 58%. Among the 329 patients, the majority were male (233, 70.8%), while the remainder were female (96, 29.2%). The mean age of the patients was 63.0 years (IQR: 54.0-71.0), with the majority (67.5%) educated to a junior high school level or below. Compared to those without poor sleep quality, CHF patients with comorbid poor sleep quality showed statistically significant differences in age (*Z* = -8.059, *P* < 0.001), smoking history (*Z* = 8.073, *P* = 0.004), atrial fibrillation (*χ^2^* = 16.821, *P* < 0.001) and anxiety symptoms (*χ^2^* = 9.274, *P* = 0.01) (**Table 1**).

**Table 1:** Baseline characteristics and differences in MCI (n=329).

Variables	Total	Non-MCI(n=138)	MCI(n=191)	Z/χ^2^	P-value
Age, years	63.00(54.00, 71.00)	55.00(48.00, 63.25)	67.00(61.00, 74.00)	-8.059	**<0.001**
Sex				2.373	0.123
Male	233(70.8)	104(75.4)	129(67.5)		
Female	96(29.2)	34(24.6)	62(32.5)		
BMI, kg/m²	24.39(21.88, 26.96)	24.76(22.66, 28.04)	23.87(21.48, 25.91)	-3.115	**0.002**
Educational level				-11.329	**0.01**
Primary school or below	101(30.7)	37(26.8)	64(33.5)		
Junior high school	121(36.8)	53(38.4)	68(35.6)		
Vocational school or high school	62(18.8)	20(14.5)	42(22.0)		
College or above	45(13.7)	28(20.3)	17(8.9)		
Marital status				5.243	**0.022**
Unmarried/divorced/widowed	35(10.6)	21(15.2)	14 (7.3)		
Married	294(89.4)	117(84.8)	177(92.7)		
Smoking history				8.073	**0.004**
No	248(75.4)	93(67.4)	155(81.2)		
Yes	81(24.6)	45(32.6)	36(18.8)		
Drinking history				8.174	**0.004**
No	250(76.0)	94(68.1)	156(81.7)		
Yes	79(24.0)	44(31.9)	35(18.3)		
NYHA class				0.592	0.744
II	138(41.9)	60(43.5)	78(40.8)		
III	125(38.0)	53(38.4)	72(37.7)		
IV	66(20.1)	25(18.1)	41(21.5)		
Hypertension				0.512	0.474
No	145(44.1)	64(46.4)	81(42.4)		
Yes	184(55.9)	74(53.6)	110(57.6)		
Diabetes				1.085	0.298
No	238(72.3)	104(75.4)	134(70.2)		
Yes	91(27.7)	34(24.6)	57(29.8)		
Stroke				3.256	0.071
No	203(61.7)	93(67.4)	110(57.6)		
Yes	126(38.3)	45 (32.6)	81 (42.4)		
Atrial Fibrillation				16.821	**<0.001**
No	138(41.9)	76(55.1)	62(32.5)		
Yes	191(58.1)	62(44.9)	129(67.5)		
LVEF				2.904	0.234
≤40%	99(30.1)	47(34.1)	52(27.2)		
41%-49%	54(16.4)	18(13.0)	36(18.8)		
≥50%	176(53.5)	73(52.9)	103(53.9)		
Depressive symptoms				27.423	**<0.001**
No	98(29.8)	62(44.9)	36(18.8)		
Mild	142(43.2)	43(31.2)	99(51.8)		
Moderate	67(20.4)	26(18.8)	41(21.5)		
Severe	22(6.7)	7(5.1)	15(7.9)		
Anxiety symptoms				9.274	**0.01**
No	106(32.2)	57(41.3)	49(25.7)		
Mild	132(40.1)	46(33.3)	86(45.0)		
Moderate	91(27.7)	35(25.4)	56(29.3)		
Hemoglobin, g/L	133.00(118.04,145.00)	135.50(122.53,146.25)	131.00(113.00,143.00)	-2.698	**0.007**
Hs-CRP, mg/L	2.80(0.80,10.00)	3.06(0.80,9.91)	2.60(0.80,10.00)	-0.147	0.883
Total Cholesterol, mmol/L	3.89(3.27,4.81)	3.96(3.29,4.83)	3.82(3.24,4.70)	-1.478	0.14
Triglycerides, mmol/L	1.22(0.92,1.78)	1.36(0.95,2.07)	1.16(0.88,1.58)	-3.122	**0.002**
Serum Creatinine, µmol/L	92.00(73.50,123.00)	91.50(70.00,119.00)	93.00(77.00,131.00)	-1.637	0.102
Alanine Aminotransferase, U/L	21.00(14.00,32.00)	23.00(16.00,36.50)	19.00(13.00,31.00)	2.923	**0.003**
Aspartate Aminotransferase, U/L	23.00(18.00,32.00)	23.00(18.00,31.00)	22.00(18.00,33.00)	-0.115	0.908
NT-proBNP, pg/mL	1695.00(467.00,4855.00)	1831.00(360.25,4105.00)	1674.00(530.00,6131.00)	-1.244	0.214
Nutritional risk	3.00(2.00,4.00)	2.00(1.00,3.00)	3.00(2.00,5.00)	-7.51	**<0.001**
PSQI	9.00(7.00,12.00)	8.00(6.00,10.00)	10.00(8.00,13.00)	**-5.511**	**<0.001**

aseline characteristics and differences in MCI(n=329).

Bold values indicate that *P*-values are <0.5.

### Current status of sleep quality in patients with CHF

In this study, PSQI scores ranged from 2 to 19, with a median total score of 9.00 (IQR: 7.00–12.00). A total of 70.52% of the patients were classified as having poor sleep quality (PSQI > 7), among whom the median score was 10.00 (IQR: 9.00–12.00). Detailed scores for all PSQI components are presented in **Table 2**. The most severely impaired component was daytime dysfunction, followed by sleep duration.

**Table 2:** Current status of sleep quality in patients with CHF(n=329).

Scales and dimensions	Score range	Median (IQR)	Minimum	Maximum
Total score of PSQI	0-21	9.00(7.00,12.00)	2	19
Subjective sleep quality	0-3	1.00(1.00,2.00)	1	3
Sleep latency	0-3	1.00(1.00,2.00)	0	3
Sleep duration	0-3	2.00(1.00,2.00)	0	3
Habitual sleep efficiency	0-3	1.00(1.00,3.00)	0	3
Sleep disturbance	0-3	1.00(1.00,1.00)	0	2
Use of sleep medication	0-3	0.00(0.00,0.00)	0	3
Daytime dysfunction	0-3	2.00(1.00,3.00)	0	3
PSQI ≤ 7(n=97,29.48%)	0-21	6.00(5.00,7.00)	2	7
PSQI>7(n=232,70.52%)	0-21	10.00(9.00,12.00)	8	19

The seven components of Pittsburgh Sleep Quality Index include subjective sleep quality, sleep latency, sleep duration, habitual sleep efficiency, sleep disturbance, use of sleep medication and daytime dysfunction.

IQR, interquartile range.

### Correlation between MCI and sleep quality in patients with CHF

The results indicated a positive correlation between MCI and overall sleep quality (*r* = 0.322, *P* < 0.01). Furthermore, MCI was significantly associated with subjective sleep quality, sleep latency, sleep duration, and habitual sleep efficiency. The corresponding correlation coefficients for each dimension are presented in **Table 3**. In contrast, no significant correlations were observed between MCI and sleep disturbance, the use of sleep medications, and daytime dysfunction (all *P* > 0.05).

**Table 3:** Correlation between MCI and sleep quality in patients with CHF(n=329).

Variables	Subjective sleep quality	Sleep latency	Sleep duration	Habitual sleep efficiency	Sleep disturbance	Use of sleep medication	Daytime dysfunction	Total score of PSQI	MCI
Subjective sleep quality	1								
Sleep latency	0.631**	1							
Sleep duration	0.498**	0.466**	1						
Habitual sleep efficiency	0.478**	0.576**	0.595**	1					
Sleep disturbance	0.393**	0.251**	0.177**	0.154**	1				
Use of sleep medication	0.214**	0.242**	0.113**	0.128**	0.007**	1			
Daytime dysfunction	-0.039	-0.005	0.010	0.002	-0.013	-0.03	1		
Total score of PSQI	0.688**	0.715**	0.652**	0.709**	0.410**	0.259**	0.246**	1	
MCI	0.227**	0.252**	0.241**	0.257**	-0.015	0.070	-0.033	0.322**	1

**P < 0.01.

### Risk factors for MCI in patients with CHF

Stratified binary logistic regression analysis was conducted with the occurrence of MCI as the dependent variable to identify independent risk factors. Model 1 included covariates such as demographic characteristics and medical history, while Model 2 added the total PSQI score to evaluate the independent role of sleep quality. The model fit indices demonstrated a good and improving fit across models. The -2LL decreased from 287.48 in Model 1 to 273.95 in Model 2, indicating a better fit with the addition of the sleep quality variable. The LRT confirmed that this improvement was statistically significant (Δχ² (1) = 13.53, *P* < 0.001). Furthermore, the explanatory power of the model increased substantially, with the Nagelkerke *R*² rising from 0.518 in Model 1 to 0.551 in Model 2, meaning that the full model (Model 2) explained 55.1% of the variance in MCI status. In the final adjusted model (Model 2), several factors were identified as statistically significant independent predictors of MCI: older age (*OR* = 1.066, 95% *CI*: 1.036-1.097, *P* < 0.001), lower educational attainment (all categories *vs*. reference, *P* < 0.05), higher nutritional risk (*OR* = 1.798, 95% *CI*: 1.464-2.208, *P* < 0.001), and poorer sleep quality (*OR* = 1.257, 95% *CI*: 1.107-1.428, *P* < 0.001). Specifically, for each one-point increase in the PSQI total score (indicating worse sleep quality), the odds of having MCI increased by 25.7% (**Table 4**).

**Table 4:** Hierarchical regression analysis regarding predictors of MCI in patients with CHF(n=329).

Variables	Model1			Model 2		
	*B*	P-value	*OR (95%CI)*	*B*	P-value	*OR (95%CI)*
Age, years	0.072	<0.001	1.075 (1.045, 1.106)	0.061	<0.001	1.066 (1.036, 1.097)
Educational level						
Primary school or below	1.31	0.011	3.706 (1.344, 10.217)	1.248	0.018	3.485 (1.239, 9.801)
Junior high school	1.552	0.003	4.720 (1.722, 12.935)	1.452	0.006	4.271 (1.513, 12.053)
Vocational school or high school	1.486	0.01	4.419 (1.426, 13.690)	1.596	0.008	4.932 (1.525, 15.954)
Nutritional risk	0.567	<0.001	1.763 (1.443, 2.156)	0.587	<0.001	1.798 (1.464, 2.208)
Total score of PSQI	–	–	–	0.229	<0.001	1.257 (1.107, 1.428)
Model Fit Indices						
-2LL	287.478			273.95		
χ2(df)	160.04 (19)	<0.001		173.57(20)	<0.001	
Δχ2(df)				13.53(1)	<0.001	
Cox-Snell R²	0.385			0.41		
Nagelkerk R²	0.518			0.551		

Coding information: Education level: 1= Primary school or below, 2= Junior high school, 3= Vocational school or high school, 4= College or above.

B, unstandardized coefficients.

## Discussion

This study revealed a 58% prevalence of MCI in patients with CHF, a result consistent with the report by Xu Chengyang et al. (37) but lower than that reported by Karen K et al. (38). This high incidence rate may be attributed to differences such as the characteristics of the study population and the clinical environment. The patients with CHF included in this study were mainly aged and with low education level. Both advanced age and low educational level are well-established, non-modifiable risk factors for cognitive impairment. In the present study, a considerable burden of depressive and anxiety symptoms, alongside poor sleep quality, constituted prominent clinical features among the patients. Existing evidence (39) suggests that these factors may exacerbate cognitive decline through multiple pathways, including dysfunction of the hypothalamic-pituitary-adrenal axis, disruption of sleep architecture, and activation of chronic inflammatory processes. Although their association with cognitive impairment did not reach statistical significance in our dataset, these mechanisms have been well-documented in the literature, and their potential clinical relevance warrants attention. In addition, CHF often triggers a series of pathological alterations, such as cerebral hypoperfusion, thrombogenesis, systemic inflammatory responses, and protein toxicity, which collectively contribute to the development of MCI (40, 41).

MCI is recognized not only for its detrimental effects on quality of life and medication adherence but also as an independent risk factor for mortality among patients with CHF (42). Although cognitive training was shown by Davis et al. to improve knowledge levels in CHF patients with MCI, it did not significantly enhance self-care behaviors or reduce rehospitalization risk (43). A randomized clinical trial in Canada (44) indicated that combined aerobic-resistance exercise and computerized cognitive training can significantly improve overall cognitive function. Greater cognitive impairment is associated with poorer self-care, higher rehospitalization and mortality rates, and worse prognoses (45). In clinical practice, it is recommended to conduct MCI screening for patients with CHF as early as possible to identify cognitive decline at an early stage and take targeted intervention measures to effectively delay cognitive decline, improve cognitive status and enhance the quality of life.

Moreover, this study further confirmed a significant positive correlation between MCI and poor sleep quality in patients with CHF. The cross-sectional nature of our study precludes any causal inference regarding the relationship between sleep quality and MCI. While our hierarchical regression models identified poor sleep quality as a significant independent risk factor, the temporal sequence of this association remains unclear. It is biologically plausible that the relationship is bidirectional: chronic poor sleep quality may contribute to cognitive impairment through mechanisms such as impaired memory consolidation and increased neuroinflammation (46). Conversely, MCI itself could lead to poor sleep quality through dysregulation of the sleep-wake cycle, anxiety related to cognitive symptoms, or other neuropathological changes (47). Poor sleep quality may act as a predisposing factor for anxiety and depressive symptoms, which could subsequently play a mediating role in the development or progression of MCI, with underlying neurophysiological alterations potentially reinforcing these interrelationships (48). Therefore, we recommend incorporating the PSQI as part of standard admission assessments for patients with CHF, with special consideration for identified high-risk subgroups such as older adults, individuals with lower educational attainment, and those at nutritional risk. Furthermore, systematic referral mechanisms should be implemented to facilitate access to dedicated sleep specialists for patients identified with clinically sleep disorders. Moreover, clinicians should prioritize evidence-based non-pharmacological strategies such as sleep hygiene education, stimulus control therapy, and cognitive behavioral therapy for insomnia as first-line treatments (49). Future randomized controlled trials are necessary to establish the benefits of these integrated care approaches on sleep parameters and cognitive function in patients with CHF.

Similar to previous research results, our research findings indicated that MCI is closely related to age, with risk progressively increasing among older individuals (50). The progression of age-related cognitive decline is significantly accelerated in patients with CHF (51). Age-related cognitive decline emerges through multiple interconnected degenerative processes, including compromised cerebrovascular autoregulation, neuronal loss, white matter lesions, and sustained neuroinflammation, which together undermine cerebral integrity (52). The persistent cerebral hypoperfusion resulting from reduced cardiac output interacts synergistically with the inherent vascular regulatory deficiencies of the aging brain, collectively compromising the structural and functional integrity of brain regions critical for cognition (40). This mechanism of heart-brain interaction makes elderly patients with heart failure an extremely high-risk group for MCI. Therefore, it is recommended to implement enhanced cognitive screening and multidisciplinary management for elderly patients with CHF, integrating HF treatment with cognitive protection strategies. This includes adapting CHF therapy to incorporate cerebroprotective objectives, optimizing blood pressure control to maintain cerebral perfusion pressure, and prioritizing medications with demonstrated neuroprotective properties.

Furthermore, this study confirmed that low educational level is significantly associated with an increased risk of MCI. Education serves as a form of cognitive reserve and is a recognized protective factor for cognitive function (53). Meta-analyses have consistently shown that higher educational levels correlate with better cognitive outcomes and reduced prevalence of MCI (54). Individuals with lower educational levels often exhibit diminished cognitive reserve, which can constrain their ability to compensate for neurodegenerative changes associated with aging or disease (55). Education fosters neuroplasticity and enhances neural efficiency, promoting the development of adaptive cognitive strategies that enable functional compensation for underlying brain pathology (56). Additionally, lower educational level is often linked to limited health literacy, which can impede effective disease self-management and increase cognitive load in patients already coping with CHF (57). In clinical practice, these findings underscore the value of identifying patients with low educational level as a high-risk subgroup for whom cognitive screening may be particularly beneficial. Providing health information in visually supported, plain-language formats may also help mitigate literacy-related barriers and support cognitive health in this population.

Nutritional risk was identified as a significant predictor of MCI, with underlying mechanisms involving multiple interrelated pathophysiological pathways. Key nutrients such as proteins, vitamin B12, vitamin D, and omega-3 fatty acids play critical roles in neurotransmitter synthesis, neuronal energy metabolism, and myelin integrity (58). In CHF patients, anorexia, malabsorption, and metabolic disturbances often lead to cardiac cachexia, which is closely linked to systemic inflammation (59). Subsequently, pro-inflammatory cytokines, notably TNF-α and IL-6, can traverse the blood-brain barrier, initiate neuroinflammatory processes, and ultimately damage memory-related regions (60). Inadequate levels of folate, vitamin B12, and antioxidants further impair brain maintenance and repair (61). Therefore, routine nutritional screening and early intervention are essential in CHF patients. Maintaining balanced nutrition supports both cardiac and cognitive health, highlighting diet as a key modifiable factor in multidisciplinary management strategies.

Although the associations between anxiety and depression symptoms with MCI did not reach statistical significance in our multivariate regression model, this result diverges from the established role of psychological factors reported by Najdaghi et al. (62). This lack of significance may be attributed to the demographic characteristics of our study population. Cognitive function appears to be more directly influenced by robust physiological factors such as cerebral hypoperfusion and nutritional risk, potentially masking the independent effects of psychological variables. Nevertheless, the clinical relevance of these psychological factors in patients with CHF warrants continued attention in both research and clinical practice. Persistent psychological stress can lead to hypothalamic-pituitary-adrenal (HPA) axis dysfunction, resulting in abnormal glucocorticoid levels that subsequently impair hippocampal neurogenesis and synaptic plasticity (63). Furthermore, patients with anxiety and depressive symptoms often experience poor sleep quality, social withdrawal, and reduced treatment adherence, all of which may indirectly accelerate cognitive decline (64). Therefore, integrating psychological symptom assessment into routine follow-up care for CHF patients using standardized instruments is recommended. For patients presenting with significant anxiety or depressive symptoms, appropriate psychological interventions should be considered. Maintaining awareness of patients’ psychological well-being may contribute to improved overall prognosis, consistent with comprehensive chronic disease management principles.

### Limitations

This study had several limitations: First, The cross-sectional design cannot determine the direction of causality between sleep quality and MCI. It remains unclear whether poor sleep contributes to cognitive decline, or if early neurodegenerative changes of MCI disrupt sleep patterns. Second, sleep assessment relied on the PSQI, a subjective measure that may be susceptible to recall and self-reporting biases. The absence of complementary objective sleep measurements, such as actigraphy or polysomnography. Additionally, as the study was conducted at a single-center in China, the generalizability of the findings was limited. We recommend that future research employs longitudinal cohort studies to track the influence of sleep quality on MCI incidence over time and conducts interventional studies to test causal relationships. Furthermore, such studies should expand the sampling scope and incorporate objective sleep measurements to enhance the representativeness and robustness of the findings.

## Conclusion

Poor sleep quality is an independent risk factor for MCI in patients with CHF, requiring increased clinical attention. Healthcare providers should routinely assess sleep quality and cognitive function in these patients. Individuals with poor sleep or additional risk factors receive early intervention and undergo long-term cognitive monitoring to track changes, with the aim of delaying cognitive decline and improving quality of life. Further research is recommended to examine the longitudinal trajectories of MCI and sleep quality in this population, which may help develop targeted intervention strategies.

## Data Availability

The raw data supporting the conclusions of this article will be made available by the authors, without undue reservation.

## Generative Al statement

The author(s) declare that Generative AI was used in the creation of this manuscript. The author(s) declare that Deepseek was employed exclusively to improve the language clarity, grammar, and overall readability of the English text in the creation of this manuscript. If you identify any issues, please contact us.

Any alternative text (alt text) provided alongside figures in this article has been generated by Frontiers with the support of artificial intelligence and reasonable efforts have been made to ensure accuracy, including review by the authors wherever possible. If you identify any issues, please contact us.

## References

[1] Zhang H Zheng X Huang P Guo L Zheng Y Zhang D . The burden and trends of heart failure caused by ischaemic heart disease at the global, regional, and national levels from 1990 to 2021. Eur Heart J Qual Care Clin Outcomes. (2025) 11:186–96. doi: 10.1093/ehjqcco/qcae094 39537193

[2] Khan MS Shahid I Bennis A Rakisheva A Metra M Butler J . Global epidemiology of heart failure. Nat Rev Cardiol. (2024) 21:717–34. doi: 10.1038/s41569-024-01046-6 38926611

[3] Savarese G Becher PM Lund LH Seferovic P Rosano GMC Coats AJS . Global burden of heart failure: a comprehensive and updated review of epidemiology. Cardiovasc Res. (2023) 118:3272–87. doi: 10.1093/cvr/cvac013 35150240

[4] Yan T Zhu S Yin X Xie C Xue J Zhu M . Burden, trends, and inequalities of heart failure globally, 1990 to 2019: a secondary analysis based on the global burden of disease 2019 study. J Am Heart Assoc. (2023) 12:e027852. doi: 10.1161/JAHA.122.027852 36892088 PMC10111559

[5] Virani SS Alonso A Aparicio HJ Benjamin EJ Bittencourt MS Callaway CW . Heart disease and stroke statistics-2021 update: a report from the american heart association. Circulation. (2021) 143:e254–743. doi: 10.1161/CIR.0000000000000950 PMC13036842 33501848

[6] Cheng RK Cox M Neely ML Heidenreich PA Bhatt DL Eapen ZJ . Outcomes in patients with heart failure with preserved, borderline, and reduced ejection fraction in the medicare population. Am Heart J. (2014) 168:721–30. doi: 10.1016/j.ahj.2014.07.008 25440801

[7] Savarimuthu A Ponniah RJ . Cognition and cognitive reserve. Integr Psychol Behav. (2024) 58:483–501. doi: 10.1007/s12124-024-09821-3 38279076

[8] Gorodeski EZ Goyal P . Cognitive impairment is our job too. J Cardiac Failure. (2024) 30:423–4. doi: 10.1016/j.cardfail.2024.02.004 38485294

[9] Chinese Society of Cardiology, Editorial Board of Chinese Journal of Cardiology . Chinese expert consensus on cardiovascular diseases and cognitive impairment. Chin J Cardiol. (2023) 51:455–68. doi: 10.3760/cma.j.cn112148-20230220-00096 37198116

[10] McGirr A Nathan S Ghahremani M Gill S Smith EE Ismail Z . Progression to dementia or reversion to normal cognition in mild cognitive impairment as a function of late-onset neuropsychiatric symptoms. Neurology. (2022) 98(21):e2132–9. doi: 10.1212/WNL.0000000000200256 35351783 PMC9169943

[11] Cong L Ren Y Wang Y Hou T Dong Y Han X . Mild cognitive impairment among rural-dwelling older adults in China: A community-based study. Alzheimers Dement. (2023) 19:56–66. doi: 10.1002/alz.12629 35262288 PMC10078715

[12] Zuccalà G Marzetti E Cesari M Lo Monaco MR Antonica L Cocchi A . Correlates of cognitive impairment among patients with heart failure: Results of a multicenter survey. Am J Med. (2005) 118:496–502. doi: 10.1016/j.amjmed.2005.01.030 15866252

[13] Dong Y Teo SY Kang K Tan M Ling LH Yeo PSD . Cognitive impairment in asian patients with heart failure: prevalence, biomarkers, clinical correlates, and outcomes. Eur J Heart Fail. (2019) 21:688–90. doi: 10.1002/ejhf.1442 30938010

[14] Sargent L Flattery M Shah K Price ET Tirado C Oliveira T . Influence of physiological and psychological factors on cognitive dysfunction in heart failure patients. Appl Nurs Res. (2020) 56:151375. doi: 10.1016/j.apnr.2020.151375 33280793

[15] Xu J Xiang L Zhang H Sun X Xu D Wu D . Prevalence and modifiable risk factors of cognitive frailty in patients with chronic heart failure in China: A cross-sectional study. BMC Cardiovasc Disord. (2024) 24:93. doi: 10.1186/s12872-024-03753-x 38326774 PMC10848518

[16] Kong Y Jiang C Zhou L Ye Y He L Chen Q . Analysis of clinicalcharacteristics and related factors in patients with common cardiovascular diseasescomplicated by mild cognitive impairment. Natl Med J China. (2024) 104:132–7. doi: 10.3760/cma.j.cn112137-20230812-00209 38186134

[17] Zheng T . Sleep disturbance in heart failure: A concept analysis. Nurs Forum. (2021) 56:710–6. doi: 10.1111/nuf.12566 PMC8349762 33665809

[18] Qin C Jin X Ji W Bao J . Research progress on the relationship between sleep disorders and cardiovascular diseases. Chin Heart J. (2023) 35:76–82.

[19] Formiga F Martínez-Velilla N . Insuficiencia cardíaca e insomnio: Una relación bidireccional. Rev Esp Geriatr Gerontol. (2022) 57:61–2. doi: 10.1016/j.regg.2021.12.002 35190192

[20] Sun Y Shao J Wang Y Wang J . Intervention effect of Baduanjin combined withmindfulness - based stress reduction on elderly patients with chronic heart failureand insomnia. J Med Forum. (2023) 44:100–4.

[21] Wang Q Xu S Liu F Liu Y Chen K Huang L . Causal relationship between sleep traits and cognitive impairment: A mendelian randomization study. J Evidence-Based Med. (2023) 16:485–94. doi: 10.1111/jebm.12576 38108111

[22] Xu W Tan C-C Zou J-J Cao X-P Tan L . Sleep problems and risk of all-cause cognitive decline or dementia: An updated systematic review and meta-analysis. J Neurol Neurosur Ps. (2020) 91:236–44. doi: 10.1136/jnnp-2019-321896 PMC7035682 31879285

[23] Zhang L Li T Lei Y Cheng G Liu B Yu Y . Association between sleep structure and amnesic mild cognitive impairment in patients with insomnia disorder: A case-control study. J Clin Sleep Med. (2021) 17:37–43. doi: 10.5664/jcsm.8804 32946373 PMC7849649

[24] Gao F Wei S Dang L Gao Y Gao L Shang S . Sleep disturbance is associated with mild cognitive impairment: A community population-based cross-sectional study. BMC Public Health. (2022) 22:2000. doi: 10.1186/s12889-022-14391-3 36320021 PMC9624002

[25] Chinese Society of Cardiology Chinese Medical Association . Cardiology branch of chinese medical doctors association, heart failure professional committee of chinese medical doctors association, editorial board of chinese journal of cardiology. Chinese guidelines for the diagnosis and treatment of heart failure2024. ChineseJournal Cardiol. (2024) 52:235–75. doi: 10.3760/cma.j.cn112148-20231101-00405

[26] Xiong J Qin J Gong K . Association between fear of progression and sleep quality in patients with chronic heart failure: a cross-sectional study. J Adv Nurs. (2023) 79:3082–91. doi: 10.1111/jan.15657 36978259

[27] Nasreddine ZS Phillips NA Bédirian V Charbonneau S Whitehead V Collin I . The montreal cognitive assessment, MoCA: a brief screening tool for mild cognitive impairment. J Am Geriatr Soc. (2005) 53:695–9. doi: 10.1111/j.1532-5415.2005.53221.x 15817019

[28] Yang L Tang X Zhou N Zhang M Jie Y . Application and reliability verification of the Beijing version of the Montreal Cognitive Assessment Scale in the cognitive function assessment of adult patients with obstructive sleep apnea - hypopnea syndrome (OSAHS). J Clin Otorhinolaryngology Head Neck Surg. (2018) 32:58–64. doi: 10.13201/j.issn.1001-1781.2018.01.012 29798212

[29] Buysse DJ Reynolds CF Monk TH Berman SR Kupfer DJ . The pittsburgh sleep quality index: A new instrument for psychiatric practice and research. Psychiatry Res. (1989) 28:193–213. doi: 10.1016/0165-1781(89)90047-4 2748771

[30] Liu X Tang M Hu L Wang A Wu H Zhao G . A study on the reliability and validity of the Pittsburgh Sleep Quality Index. Chin J Psychiatry. (1996) 1996(02):103–7.

[31] Spitzer RL . Validation and utility of a self-report version of PRIME-MDThe PHQ primary care study. JAMA. (1999) 282:1737. doi: 10.1001/jama.282.18.1737 10568646

[32] Bian C He X Qian J Wu W Li C . Application research on the Patient Health Questionnaire -Depression Symptom Group Scale in general hospitals. J Tongji Univ (Medical Science). (2009) 30(05):136–40.

[33] Spitzer RL Kroenke K Williams JBW Löwe B . A brief measure for assessing generalized anxiety disorder: the GAD-7. Arch Intern Med. (2006) 166:1092. doi: 10.1001/archinte.166.10.1092 16717171

[34] Zhu G Bing Y Liu L . Anxiety status and its influencing factors among elderly patients with hypertension in a district of Shanghai. Sichuan Ment Health. (2022) 35:26–30. doi: 10.11886/scjsws20210718001

[35] de Ulíbarri JI González-Madroño A de Villar NG González P González B Mancha A . CONUT: a tool for controlling nutritional status. First validation in a hospital population. Nutr Hosp. (2005) 20(1):38–45. 15762418

[36] Zhao M Song J Zhou D Huang L Zhang X Chen H . Malnutrition risk assessment and analysis of influencing factors in elderly hospitalized patients with chronic heart failure. Chin Circ J. (2023) 38:1164–70. doi: 10.3969/j.issn.1000-3614.2023.11.010

[37] Xu C Tao X Ma X Zhao R Cao Z . Cognitive dysfunction after heart disease: A manifestation of the heart-brain axis. Oxid Med Cell Longev. (2021) 2021:4899688. doi: 10.1155/2021/4899688 34457113 PMC8387198

[38] Davis KK Himmelfarb CRD Szanton SL Hayat MJ Allen JK . Predictors of heart failure self-care in patients who screened positive for mild cognitive impairment. J Cardiovasc Nurs. (2015) 30:152–60. doi: 10.1097/JCN.0000000000000130 24434832

[39] Angermann CE Ertl G . Depression, anxiety, and cognitive impairment: Comorbid mental health disorders in heart failure. Curr Heart Fail Rep. (2018) 15:398–410. doi: 10.1007/s11897-018-0414-8 30402659

[40] Park MS Kim EJ . A correlative relationship between heart failure and cognitive impairment: A narrative review. J Korean Med Sci. (2023) 38:e334. doi: 10.3346/jkms.2023.38.e334 37821090 PMC10562184

[41] Ye S Huynh Q Potter EL . Cognitive dysfunction in heart failure: Pathophysiology and implications for patient management. Curr Heart Fail Rep. (2022) 19:303–15. doi: 10.1007/s11897-022-00564-z 35962923

[42] Ni RSS Mohamed Raffi HQ Dong Y . The pathophysiology of cognitive impairment in individuals with heart failure: A systematic review. Front Cardiovasc Med. (2023) 10:1181979. doi: 10.3389/fcvm.2023.1181979 37288268 PMC10242665

[43] Davis KK Mintzer M Himmelfarb CRD Hayat MJ Rotman S Allen J . Targeted intervention improves knowledge but not self-care or readmissions in heart failure patients with mild cognitive impairment. Eur J Heart Fail. (2012) 14:1041–9. doi: 10.1093/eurjhf/hfs096 22736737

[44] Montero-Odasso M Zou G Speechley M Almeida QJ Liu-Ambrose T Middleton LE . Effects of exercise alone or combined with cognitive training and vitamin D supplementation to improve cognition in adults with mild cognitive impairment: A randomized clinical trial. JAMA Netw Open. (2023) 6:e2324465. doi: 10.1001/jamanetworkopen.2023.24465 37471089 PMC10359965

[45] Vellone E Chialà O Boyne J Klompstra L Evangelista LS Back M . Cognitive impairment in patients with heart failure: An international study. ESC Heart Fail. (2020) 7:46–53. doi: 10.1002/ehf2.12542 31854133 PMC7083494

[46] Byun E Kim J Riegel B . Associations of subjective sleep quality and daytime sleepiness with cognitive impairment in adults and elders with heart failure. Behav Sleep Med. (2017) 15:302–17. doi: 10.1080/15402002.2015.1133418 27116617

[47] Gilstrap LG Gorodeski EZ Goyal P . Heart failure and cognitive impairment: Complexity that requires a new approach. J Am Geriatr Soc. (2022) 70:1652–4. doi: 10.1111/jgs.17779 PMC9177752 35426956

[48] Mo W Liu X Yamakawa M . Prevalence of sleep disturbances in people with mild cognitive impairment: A systematic review protocol. JBI Evidence Synth. (2023) 21:2211–7. doi: 10.11124/JBIES-22-00438 37338281

[49] Cassidy-Eagle E Siebern A Unti L Glassman J O’Hara R . Neuropsychological functioning in older adults with mild cognitive impairment and insomnia randomized to CBT-I or control group. Clin Gerontol. (2018) 41:136–44. doi: 10.1080/07317115.2017.1384777 29220627

[50] Li G Toschi N Devanarayan V Batrla R Boccato T Cho M . The age-specific comorbidity burden of mild cognitive impairment: a US claims database study. Alzheimer’s Res Ther. (2023) 15:211. doi: 10.1186/s13195-023-01358-8 38057937 PMC10701954

[51] Chu G Zhang L Wang C . Analysis of influencing factors for cognitive impairment in patients with chronic heart failure. Chin J Integr Med Cardio-Cerebrovascular Dis. (2024) 22:2514–7. doi: 10.12102/j.issn.1672-1349.2024.14.003

[52] Vromen EM de Leeuw DM van Harten AC . CSF proteomic profiles related to cognitive decline in MCI A+ depend on tau levels. Brain. (2025). doi: 10.1093/brain/awaf251. PMC12677021 40676952

[53] Klee M Aho VTE May P Heintz-Buschart A Landoulsi Z Jónsdóttir SR . Education as risk factor of mild cognitive impairment: the link to the gut microbiome. J Prev Alzheimer’s Dis. (2024) 11:759–68. doi: 10.14283/jpad.2024.19 PMC11060993 38706292

[54] Lövdén M Fratiglioni L Glymour MM Lindenberger U Tucker-Drob EM . Education and cognitive functioning across the life span. Psychol Sci Public Interest. (2020) 21:6–41. doi: 10.1177/1529100620920576 32772803 PMC7425377

[55] Han X Li Y Wang J Liu X Zhang Y Dong Q . Associations between lifelong cognitive reserve, alzheimer’s disease-related plasma biomarkers, and cognitive function in dementia-free older adults: a population-based study. J Alzheimer’s Dis. (2025) 103:821–32. doi: 10.1177/13872877241306448 39791378

[56] LoBue C Denney D Hynan LS Rossetti HC Lacritz LH Hart J . Self-reported traumatic brain injury and mild cognitive impairment: increased risk and earlier age of diagnosis. J Alzheimers Dis. (2016) 51:727–36. doi: 10.3233/JAD-150895 PMC4853649 26890760

[57] Han SD Boyle PA James BD Yu L Bennett DA . Poorer financial and health literacy among community-dwelling older adults with mild cognitive impairment. J Aging Health. (2015) 27:1105–17. doi: 10.1177/0898264315577780 PMC4520748 25903976

[58] Lee KS Hong CH Cheong H-K Oh BH . Difference in nutritional risk between mild cognitive impairment group and normal cognitive function elderly group. Arch Gerontol Geriatr. (2009) 49:49–53. doi: 10.1016/j.archger.2008.04.011 18524396

[59] Liao T Gan Y Liu L Du Y Li G Guo M . The association of sleep quality with anorexia in patients with heart failure: do anxiety and depressive symptoms mediate this association. J Cardiovasc Nurs. (2025). doi: 10.1097/JCN.0000000000001248 PMC13064829 40801828

[60] Mehler PS Watters A Joiner T Krantz MJ . What accounts for the high mortality of anorexia nervosa? Int J Eat Disord. (2022) 55:633–6. doi: 10.1002/eat.23664 34997783

[61] de Wilde MC Vellas B Girault E Yavuz AC Sijben JW . Lower brain and blood nutrient status in alzheimer’s disease: Results from meta-analyses. Alzheimers Dement (N Y). (2017) 3:416–31. doi: 10.1016/j.trci.2017.06.002 PMC5651428 29067348

[62] Najdaghi S Narimani Davani D Shafie D . Predictors of poor sleep quality in heart failure patients: a cross-sectional multivariable analysis of clinical, demographic, and psychosocial factors. J Res Health Sci. (2025) 25:e00649. doi: 10.34172/jrhs.2025.184 40259652 PMC12009488

[63] Franks KH Rowsthorn E Bransby L Lim YY Chong TT-J Pase MP . Association of self-reported psychological stress with cognitive decline: a systematic review. Neuropsychol Rev. (2023) 33:856–70. doi: 10.1007/s11065-022-09567-y 36456767

[64] Yu J Rawtaer I Fam J Jiang M Feng L Kua EH . Sleep correlates of depression and anxiety in an elderly <span style=“font-variant:Small-caps;”>a sian population. Psychogeriatrics. (2016) 16:191–5. doi: 10.1111/psyg.12138 26179204

