# Correction: Association between mild cognitive impairment and sleep quality in patients with chronic heart failure: a cross-sectional study

**DOI:** 10.3389/fpsyt.2026.1913197

**Published:** 2026-07-07

**Authors:** Yanmei Gan, Tingting Liao, Yao Du, Lingfang Liu, Lan Luo, Wenhua Huang, Gaoye Li

**Affiliations:** 1 Department of Cardiovascular Medicine, The First Affiliated Hospital of Guangxi Medical University, Nanning, China; 2 Department of Radiation Oncology, The First Affiliated Hospital of Guangxi Medical University, Nanning, China

**Keywords:** chronic heart failure, mild cognitive impairment, sleep quality, sleep disorders, risk factors

In **Table 1**, the column headings “Non-MCI(n=138)” and “MCI(n=191)” were erroneously swapped, resulting in the data for the two groups being presented under the wrong headings. Additionally, the column heading “Totall” was misspelled. The corrected **Table 1** appears below. The original version of this article has been updated.

**Table 1:** Baseline characteristics and differences in MCI (n=329).

Variables	Total	Non-MCI(n=138)	MCI(n=191)	Z/χ^2^	P-value
Age, years	63.00(54.00, 71.00)	55.00(48.00, 63.25)	67.00(61.00, 74.00)	-8.059	**<0.001**
Sex				2.373	0.123
Male	233(70.8)	104(75.4)	129(67.5)		
Female	96(29.2)	34(24.6)	62(32.5)		
BMI, kg/m²	24.39(21.88, 26.96)	24.76(22.66, 28.04)	23.87(21.48, 25.91)	-3.115	**0.002**
Educational level				-11.329	**0.01**
Primary school or below	101(30.7)	37(26.8)	64(33.5)		
Junior high school	121(36.8)	53(38.4)	68(35.6)		
Vocational school or high school	62(18.8)	20(14.5)	42(22.0)		
College or above	45(13.7)	28(20.3)	17(8.9)		
Marital status				5.243	**0.022**
Unmarried/divorced/widowed	35(10.6)	21(15.2)	14 (7.3)		
Married	294(89.4)	117(84.8)	177(92.7)		
Smoking history				8.073	**0.004**
No	248(75.4)	93(67.4)	155(81.2)		
Yes	81(24.6)	45(32.6)	36(18.8)		
Drinking history				8.174	**0.004**
No	250(76.0)	94(68.1)	156(81.7)		
Yes	79(24.0)	44(31.9)	35(18.3)		
NYHA class				0.592	0.744
II	138(41.9)	60(43.5)	78(40.8)		
III	125(38.0)	53(38.4)	72(37.7)		
IV	66(20.1)	25(18.1)	41(21.5)		
Hypertension				0.512	0.474
No	145(44.1)	64(46.4)	81(42.4)		
Yes	184(55.9)	74(53.6)	110(57.6)		
Diabetes				1.085	0.298
No	238(72.3)	104(75.4)	134(70.2)		
Yes	91(27.7)	34(24.6)	57(29.8)		
Stroke				3.256	0.071
No	203(61.7)	93(67.4)	110(57.6)		
Yes	126(38.3)	45 (32.6)	81 (42.4)		
Atrial Fibrillation				16.821	**<0.001**
No	138(41.9)	76(55.1)	62(32.5)		
Yes	191(58.1)	62(44.9)	129(67.5)		
LVEF				2.904	0.234
≤40%	99(30.1)	47(34.1)	52(27.2)		
41%-49%	54(16.4)	18(13.0)	36(18.8)		
≥50%	176(53.5)	73(52.9)	103(53.9)		
Depressive symptoms				27.423	**<0.001**
No	98(29.8)	62(44.9)	36(18.8)		
Mild	142(43.2)	43(31.2)	99(51.8)		
Moderate	67(20.4)	26(18.8)	41(21.5)		
Severe	22(6.7)	7(5.1)	15(7.9)		
Anxiety symptoms				9.274	**0.01**
No	106(32.2)	57(41.3)	49(25.7)		
Mild	132(40.1)	46(33.3)	86(45.0)		
Moderate	91(27.7)	35(25.4)	56(29.3)		
Hemoglobin, g/L	133.00(118.04,145.00)	135.50(122.53,146.25)	131.00(113.00,143.00)	-2.698	**0.007**
Hs-CRP, mg/L	2.80(0.80,10.00)	3.06(0.80,9.91)	2.60(0.80,10.00)	-0.147	0.883
Total Cholesterol, mmol/L	3.89(3.27,4.81)	3.96(3.29,4.83)	3.82(3.24,4.70)	-1.478	0.14
Triglycerides, mmol/L	1.22(0.92,1.78)	1.36(0.95,2.07)	1.16(0.88,1.58)	-3.122	**0.002**
Serum Creatinine, µmol/L	92.00(73.50,123.00)	91.50(70.00,119.00)	93.00(77.00,131.00)	-1.637	0.102
Alanine Aminotransferase, U/L	21.00(14.00,32.00)	23.00(16.00,36.50)	19.00(13.00,31.00)	2.923	**0.003**
Aspartate Aminotransferase, U/L	23.00(18.00,32.00)	23.00(18.00,31.00)	22.00(18.00,33.00)	-0.115	0.908
NT-proBNP, pg/mL	1695.00(467.00,4855.00)	1831.00(360.25,4105.00)	1674.00(530.00,6131.00)	-1.244	0.214
Nutritional risk	3.00(2.00,4.00)	2.00(1.00,3.00)	3.00(2.00,5.00)	-7.51	**<0.001**
PSQI	9.00(7.00,12.00)	8.00(6.00,10.00)	10.00(8.00,13.00)	**-5.511**	**<0.001**

aseline characteristics and differences in MCI(n=329).

Bold values indicate that *P*-values are <0.5.

